# From Chromosomes to Genome: Insights into the Evolutionary Relationships and Biogeography of Old World Knifefishes (Notopteridae; Osteoglossiformes)

**DOI:** 10.3390/genes9060306

**Published:** 2018-06-19

**Authors:** Felipe Faix Barby, Petr Ráb, Sébastien Lavoué, Tariq Ezaz, Luiz Antônio Carlos Bertollo, Andrzej Kilian, Sandra Regina Maruyama, Ezequiel Aguiar de Oliveira, Roberto Ferreira Artoni, Mateus Henrique Santos, Oladele Ilesanmi Jegede, Terumi Hatanaka, Alongklod Tanomtong, Thomas Liehr, Marcelo de Bello Cioffi

**Affiliations:** 1 Departamento de Genética e Evolução, Universidade Federal de São Carlos (UFSCar), Rodovia Washington Luiz Km. 235, C.P. 676, São Carlos, SP 13565-905, Brazil; felipe_barby@hotmail.com (F.F.B.); bertollo@ufscar.br (L.A.C.B.); srmaruyama@gmail.com (S.R.M.); ezekbio@gmail.com (E.A.d.O.); hterumi@yahoo.com.br (T.H.); 2 Laboratory of Fish Genetics, Institute of Animal Physiology and Genetics, Czech Academy of Sciences, Rumburská 89, 277 21 Liběchov, Czech Republic; rab@iapg.cas.cz; 3 Institute of Oceanography, National Taiwan University, Roosevelt Road, Taipei 10617, Taiwan; microceb@hotmail.com; 4 Institute for Applied Ecology, University of Canberra, Canberra, ACT 2617, Australia; Tariq.Ezaz@canberra.edu.au; 5 Diversity Arrays Technology, University of Canberra, Bruce, Australian Capital Territory, Canberra, ACT 2617, Australia; zej@diversityarrays.com; 6 Departamento de Biologia Estrutural, Molecular e Genética, Universidade Estadual de Ponta Grossa, Ponta Grossa, PR 84030-900 Brazil; rfartoni@gmail.com (R.F.A.); mhsantos@uepg.br (M.H.S.); 7 Department of Fisheries and Aquaculture, Adamawa State University, P.M.B. 25 Mubi. Adamawa State, Nigeria; jegedeio@yahoo.co.uk; 8 Toxic Substances in Livestock and Aquatic Animals Research Group, KhonKaen University, Muang, KhonKaen 40002, Thailand; tanomtong@hotmail.com; 9 Institute of Human Genetics, University Hospital Jena, 07747 Jena, Germany; Thomas.Liehr@med.uni-jena.de

**Keywords:** Out-of-India, Lemurian stepping-stones, Notopteridae, Osteoglossiformes, fluorescent in situ hybridization (FISH), DArTseq NGS, chromosomal evolution

## Abstract

In addition to its wide geographical distribution, osteoglossiform fishes represent one of the most ancient freshwater teleost lineages; making it an important group for systematic and evolutionary studies. These fishes had a Gondwanan origin and their past distribution may have contributed to the diversity present in this group. However, cytogenetic and genomic data are still scarce, making it difficult to track evolutionary trajectories within this order. In addition, their wide distribution, with groups endemic to different continents, hinders an integrative study that allows a globalized view of its evolutionary process. Here, we performed a detailed chromosomal analysis in Notopteridae fishes, using conventional and advanced molecular cytogenetic methods. Moreover, the genetic distances of examined species were assessed by genotyping using diversity arrays technology sequencing (DArTseq). These data provided a clear picture of the genetic diversity between African and Asian Notopteridae species, and were highly consistent with the chromosomal, geographical, and historical data, enlightening their evolutionary diversification. Here, we discuss the impact of continental drift and split of Pangea on their recent diversity, as well as the contribution to biogeographical models that explain their distribution, highlighting the role of the Indian subcontinent in the evolutionary process within the family.

## 1. Introduction

The monophyletic order Osteoglossiformes is one of the most iconic and ancient (Early Triassic) group of freshwater teleosts, and it is nowadays restricted to tropical regions of South America, Africa, Asia, and Australia [1,2,3]. The intercontinental distribution of the Osteoglossiformes, combined with their intolerance to salinity and rich fossil record, attracted a lot of attention in biogeography to determine the contribution of tectonic-mediated vicariant events relative to marine or geodispersal events [4].

In particular, geographical distribution of the osteoglossiform family Notopteridae “Old World knifefishes” [5] in Africa, India, and Southeast Asia represents one of the long-standing challenging problems of freshwater biogeography at the intercontinental scale [6]. The Notopteridae include four genera, namely *Chitala*, *Notopterus*, *Xenomystus*, and *Papyrocranus*, and about ten species [7]. While *Chitala* and *Notopterus* are endemic to the Indian region and Southeast Asia (i.e., Sundaland and Indochina), *Papyrocranus* and *Xenomystus* are endemic to West and Central Africa [7]. All species are restricted to freshwaters, although [7] reported that the bronze featherfish, *Notopterus notopterus*, may occasionally stand low levels of salinity. *Chitala* currently comprises six valid species, while *Papyrocranus* includes two species, and *Xenomystus* and *Notopterus* are each monospecific [7,8]. Recent phylogenetic studies strongly support the monophyly of Notopteridae, and divide it into two clades: one African comprising *Xenomystus* and *Papyrocranus* (subfamily Xenomystinae), and one Asian clade with *Notopterus* and *Chitala* (subfamily Notopterinae) [8,9,10]. The earliest notopteroid fossil is *Palaeonotopterus greenwoodi* from a Cenomanian outcrop in [8,9,10] North Africa. However, the phylogenetic position of this fossil is still uncertain [11,12], but it conclusively marks the early presence of Notopteridae in Africa. None of the previous molecular studies, however, included the Indian *Chitala chitala*, the type species of the genus *Chitala* [7].

Despite that the higher-level phylogeny of the Notopteridae is well resolved and well supported, the biogeography of this family is still debated, mainly because of discrepancies in different molecular dating estimations. Depending on the inferred diversification timescale, there are some competing biogeographical hypotheses that have been suggested to explain the Africa–Asia distribution of Notopteridae fishes. According to [10], we have two possibilities: (1) vicariant hypothesis states that the divergence between Asian Notopterinae and African Xenomystinae was caused by the fragmentation between Africa and India/Madagascar, and (2) geodispersal hypothesis states that a land bridge between Africa and, either Eurasia or India, was used by the ancestors of Notopterinae to colonize Asia. This geodispersal event could have happened in two different moments: before the complete Pangea separation into Laurasia and Gondwana, approximately 160 Mya [13], or in a recent transcontinental migration when or after the connection between the African continents and Eurasia landmasses, as proposed by [14], through closure of the Eastern Mediterranean seaway in the early Miocene (ca. 20 Mya) [15]. Once the members of the Osteoglossomorpha are generally considered to be primary freshwater fishes [16], a transmarine dispersal hypothesis seems to be unlikely. The fossil record of Notopteridae in Asia, including the Indian region, is only partially informative, since the oldest fossils assigned to the family Notopteridae are otoliths of Cretaceous/Paleogene age were excavated from India. Their precise phylogenetic position is unresolved, although they probably belong to stem species of Notopteridae. The oldest (and only) fossil assignable to the crown group Notopterinae is a fossil of *Notopterus* unearthed from Sumatra that is, possibly, as old as the Eocene [17]. The age of this fossil provides a corresponding minimum age for the presence of Notopterinae in Asia, in such a way that a transmarine dispersal hypothesis seems to be unlikely.

Therefore, these remaining questions should be re-examined using multivariate approaches. In this line, karyotype investigations were coupled with analysis of genetic distance based on high-resolution sequencing data, and biogeographic analyses may provide further powerful insight into such problems. Although it constitutes a particular model for biogeographic and evolutionary studies [4,6,18], the cytotaxonomy of representatives within the order Osteoglossiformes is still insufficiently known, with most of the available data predating the development of modern molecular cytogenetic approaches [19,20]. These traditional data are restricted to the determination of diploid chromosome numbers (2n) and the basic karyotype descriptions of several representatives of all main Osteoglossiformes’ lineages. Despite their intrinsic limitation, such data have already demonstrated significant karyotype diversity among lineages, with an absence of heteromorphic sex chromosomes in most species [19]. To date six notopterid species were studied using only conventional cytogenetic analyzes (Table 1).

Here, we performed the first detailed chromosomal analysis of seven Notopteridae species, including the Indian *C. chitala* the latter not examined in previous studies, using conventional and advanced molecular cytogenetic methods. Then, we compared our cytogenetic results in using a powerful genotyping by high-throughput sequencing technology. All these data, along with previous genetic results based on few molecular markers, highlight many aspects of the evolution of the Old-World knifefishes. In particular, they help to characterize the genetic diversification between the African and Asian Notopteridae species, and the consequence of their geographic distribution.

## 2. Materials and Methods

### 2.1. Animals, Chromosome Preparations, and Bandings

**Figure 1:**
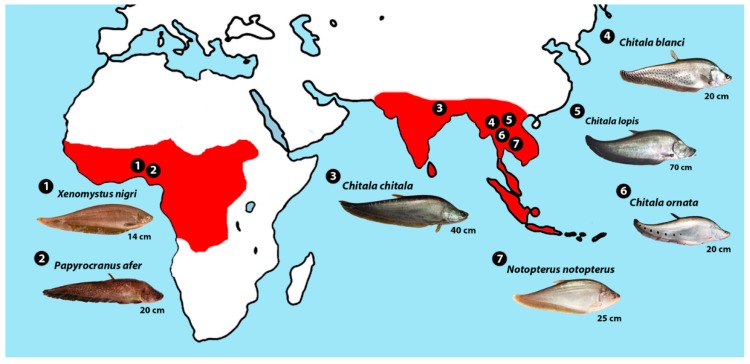
Map showing the origins of the specimens of seven notopterid species examined in this study. Localities 1 and 2 in Nigeria, 3 in India, and 4 to 7 in Thailand. The geographic distribution of the living species of Notopteridae is shown in red.

Representatives of seven species of Notopteridae were collected in various African and Southeastern Asian river basins (Figure 1; Table 2) and the individuals were deposited in the fish collection of the Museu de Zoologia, Universidade de São Paulo (vouchers 20557 and 20557). The experiments followed ethical and anesthesia conducts, in accordance with the Ethics Committee on Animal Experimentation of the Universidade Federal de São Carlos (Process number CEUA 1926260315).

Mitotic chromosomes were prepared directly from anterior kidney cells after in vivo colchicine treatment of the specimens, according to [30]. Constitutive heterochromatin (C-banding), Ag-Nucleolar Organizer Regions (Ag-NORs) stained by silver nitrate, and counterstain enhanced fluorescence (Chromomycin A3—CMA3) were performed following protocols described in [31,32,33], respectively.

### 2.2. Probes and Fluorescence In Situ Hybridization.

Two tandem-arrayed DNA sequences isolated from the genome of the species *Hoplias malabaricus* (Erythrinidae) were used as probes. The first probe contained a 5S ribosomal DNA (rDNA) repeat copy, and included 120 base pairs (bp) of the 5S rRNA transcribed gene and 200 bp of the non-transcribed spacer (NTS) sequence [34]. The second probe corresponded to a 1400 bp segment of the 18S rRNA gene obtained from nuclear DNA using polymerase chain reaction (PCR) [35]. The 5S and 18S rDNA probes were cloned into plasmid vectors and propagated in DH5α *Escherichia coli* competent cells (Invitrogen, San Diego, CA, USA).

The 18S and 5S rDNA probes were labelled with biotin-16-dUTP and digoxigenin-11-dUTP, respectively, using nick translation according to the manufacturer’s recommendations (Roche, Mannhein, Germany). The microsatellite motifs (CA)_15_, (GA)_15_, (CAA)_10_, and (CGG)_10_, used as probes for analysis of repetitive elements organization, were synthesized as described by [36]. These sequences were directly labelled with Cy3 at the 5′ termini during synthesis by Sigma (St. Louis, MO, USA). The telomeric probe (TTAGGG)n was generated by PCR (PCR DIG-Probe Synthesis Kit, Roche) in the absence of a template, using (TTAGGG)5’ and (CCCTAA)5’ as primers [37].

Fluorescence in situ hybridization (FISH) was performed as described in [38]. Signal detection was performed using avidin-Fluorescent isothiocyanate (FITC) for 18S rDNA probe, and anti-digoxigenin–rhodamine for 5S rDNA and (TTAGGG)_n_ probes. A final wash was performed at room temperature in 4× Saline-Sodium Citrate Tween (SSCT) for 5 min. Finally, the chromosomes were counterstained with 4′,6-diamidino-2-phenylindole (DAPI) (1.2 μg/mL), and mounted in antifade solution (Vector, Burlingame, CA, USA).

### 2.3. Image Processing

At least 30 metaphase spreads per individual were analyzed to confirm the 2n, karyotype structure, and results of FISH experiments. Images were captured using an Olympus BX50 epifluorescence microscope (Olympus Corporation, Ishikawa, Japan) with CoolSNAP camera (Vision Systems GmbH, Puchheim, Germany), and processed using Image Pro Plus 4.1 software (Media Cybernetics, Silver Spring, MD, USA). Chromosomes were classified metacentric (m), submetacentric (sm), subtelocentric (st), or acrocentric (a), according to [39].

### 2.4. DNA Extraction and Genotyping by Sequencing

DNA extraction was performed according to [40]. Genotyping by diversity arrays technology sequencing (DArTseq) was performed at DArT Pty Ltd. (University of Canberra, Australia), following the protocols described by [41]. For all species (except from *Xenomystus nigri*), two individuals (one male and one female) were used in the experiments. A combination of PstI and SphI enzymes was used to construct the libraries using methods described by [42], QCed and sequenced on the Illumina Hiseq2500 next generation sequencer (Illumina, San Diego, CA, USA). PstI and SphI are six base cutter targeting AG and GC rich regions and indirectly target gene rich regions of the genome. This enzyme combination was selected during the optimization steps, since they provided an average read depth as well sufficient quality markers for the analysis. Two libraries were constructed for each DNA sample and the whole process of data generation was done in full technical replication (from digestion/ligation step to marker calling). Approximately 2.5 million sequences were used per sample to produce marker data. Markers were extracted using DArT PL’s proprietary analytical pipeline which, in addition to allele calling and marker data metadata reporting, evaluates consistency of allele calling among the technical replicates.

Single-nucleotide polymorphisms (SNPs) and SilicoDArTs markers were extracted from the sequences of genomic representations (libraries). SilicoDArTs, which represent presence/absence of specific restriction fragment in genomic representations were scored as “1” for the “present” allele, and “0” for absence of the fragment/sequence. SNPs were scored in “two row” format—each row representing a specific allele at the SNP locus. The absence of the allele was scored “0” and “1” was reported for presence of the allele (Table S1) [43]. Minimum polymorphism information content (PIC), which is a function of minor allele frequency, was set at 0.02 for both reference and SNP allele. The average values were 0.242 and 0.245 respectively. For read depth, the threshold for reference allele and SNP allele were set at 2 and 1.5, respectively. The average values were 79.9 and 70.3 respectively. Finally, average reproducibility threshold was set at 93.0%, and average reproducibility was 99.9%.

### 2.5. Analysis of Genetic Diversity between Species

From the filtered SNP DArTseq data matrix, a pairwise genetic similarity matrix, based on [44], was computed and employed for genetic diversity analyses using R packages. Principal component analysis (PCA) was performed with FactorMineR [45], while hierarchical clustering analysis with *p*-values (approximately unbiased (AU) *p*-value and bootstrap probability (BP) value) was performed with pvclust [46] using Euclidean distance.

We then reconstructed the relationships among notopterid species using the DArTseq data associated with chromosomal characters. The analyses were performed at MrsBayes v. 3.2 program [47] using two separated datasets (chromosomal and DArTseq). The first six inputs in the matrix considered the chromosomal information, coded as in the Table S2. The second partition corresponded to the information 7 to 1543 from the DArTSeq data. The states of DArTseq characters were coded as zero, when no difference from the comparison data was found and one when SNP’s had differences from this dataset. We used the standard model, since we do not have information of the mutation rate from the dataset. The line “Format datatype = mixed (Standard: 1–6, Standard: 7–1543)” was used to generate the input data matrix. The Markov Monte Carlo Chain (mcmc) was set to two billion generations with sample frequency of 10,000 and 500,000 burn-in. This analysis was performed two times to reach the standard deviation below 0.05 (0.02 from this work) and posterior probability 1.000810. The cladogram generated was edited in the FigTree v.1.4.3 program [48].

### 2.6. Feature Annotation of Allele Sequences

FASTA file containing 1537 SNP allelic sequences (i.e., reference allele sequences were excluded), all of them presenting 69 nucleotides in length, were used for basic local alignment search tool (BLAST) [49] searches applying BLAST-NorBLAST-X algorithms implemented into in-house programs written in Visual Basic [50]. Allele sequences were compared to three collections of Teleostei sequences from RefSeq/NCBI database [51]: Teleostei messenger RNA (mRNA) (373,153 mRNA sequences from six species), Osteoglossiformes Nucleotide (48.195 genomic/mRNA sequences from 23 species), and Osteoglossiformes protein (41,731 protein sequences from 239 species). BLAST searches used a cut-off *E*-value (Expected value) lower than 0.1. Functional classification of allele sequences was manually curated based on description of best matches against the three RefSeq collections above. All BLAST results were plotted in a hyperlinked Excel spreadsheet (Table S3), which is best visualized using Windows OS.

## 3. Results

### 3.1. Karyotypes and Chromosome Bandings

**Figure 2:**
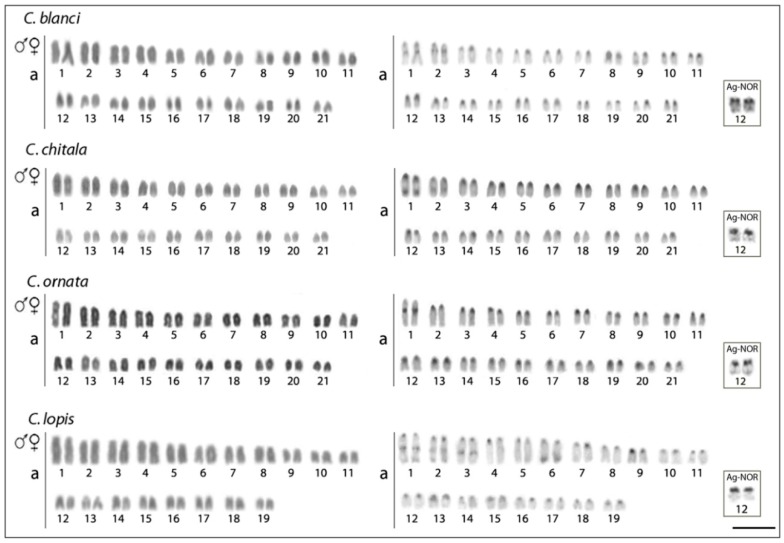
Karyotypes of four *Chitala* species analyzed after conventional Giemsa staining (**left**) and C-banding (**right**) procedures. The Ag-nucleolar organizer regions (Ag-NORs) pairs are highlighted in boxes. Scale bar = 5 µm.

**Figure 3:**
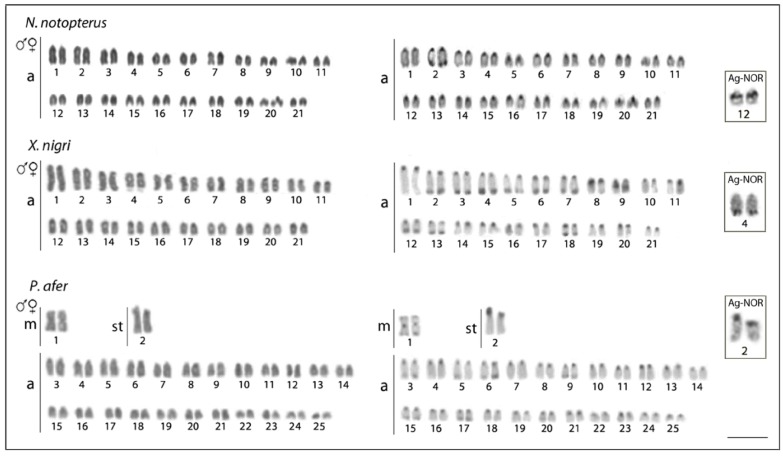
Karyotypes of *Notopterus notopterus*, *X. nigri*, and *Papyrocranus afer* analyzed after conventional Giemsa staining and C-banding procedures. The Ag-NOR pairs are highlighted in the boxes. Scale bar = 5 µm.

**Figure 4:**
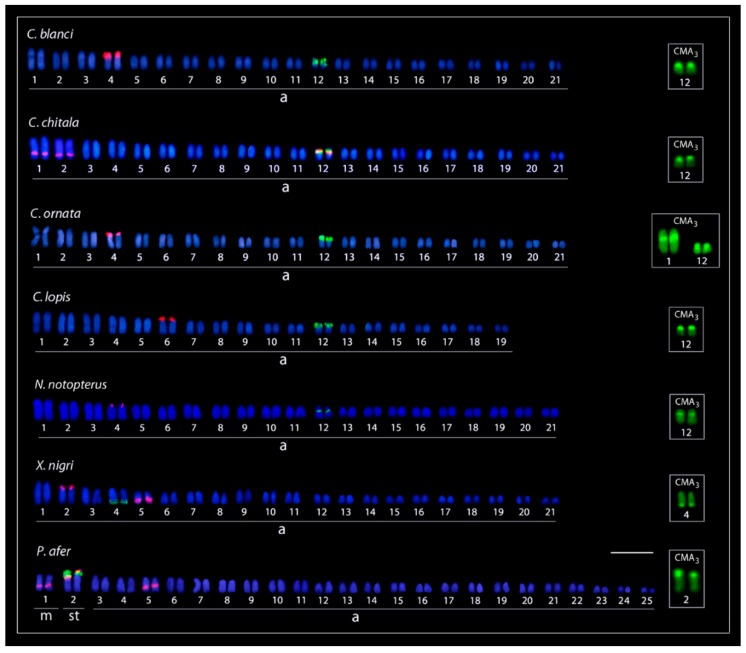
Karyotypes of seven species of Notopteridae analyzed after double fluorescent in situ hybridization (FISH) experiments with 18S (green) and 5S ribosomal DNAs (rDNAs) (red) as probes. The chromosomes evidencing GC-rich regions after Chromomycin A_3_ staining are highlighted in boxes. Scale bar = 5 µm.

The results of cytogenetic investigation of seven notopterid species are summarized in Table 1. We did not observe any karyotype differences between males and females in all species. Briefly, five species have 2n = 42 and karyotypes composed of only by acrocentric chromosomes and a NF = 42. *Chitala lopis* has 2n = 38 and a karyotype composed of chromosomes exclusively by acrocentric chromosomes (NF = 38) and *Papyrocranus afer* has 2n = 50 and a karyotype composed of 2m + 2sm + 46a chromosomes (NF = 54). C-positive heterochromatic bands were observed in the centromeric region of all chromosomes in all species, and telomeric bands in the first pair (Figure 2 and Figure 3). The karyotypes of *C. blanci* and *C. lopis* possessed interstitial bands on the two largest chromosome pairs, in addition, that of *C. lopis* had interstitial bands on the third pair and telomeric bands on the third and sixth pair of chromosomes. A single Ag-NOR site in the pericentromeric region of chromosome pair No. 12 was detected in karyotypes of all Asian species (i.e., species of *Chitala* and *Notopterus*) while in the African species, *X. nigri* and *P. afer*, they were located on the q- and p-arms of chromosomes No. 4 and 2, respectively. Single CMA_3_+ bands corresponded to the Ag-NOR sites in karyotypes of all species, except *C. ornata*, where an additional interstitial band was observed on the first chromosome pair (Figure 4).

### 3.2. Telomere (TTAGGG)n and Ribosomal DNA (5S and 18S Ribosomal DNA) Sequence Mapping

**Figure 5:**
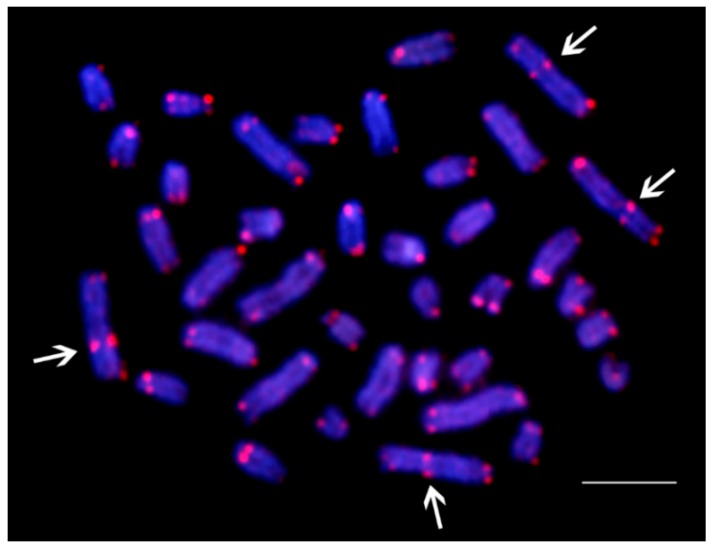
Metaphase plate of *C. lopis* hybridized with telomeric (TTAGGG)_n_ probe. Arrows indicate interstitial telomeric site (ITS) in four chromosomes. Scale bar = 5 µm.

The (TTAGGG)_n_ repeats showed the expected hybridization signals on telomeres of all species (data not shown). The only exception was the karyotype of *C. lopis* which had two ITS (interstitial telomeric sites) in the first and third chromosomal pairs (Figure 5).

The 18S rDNA positive signals corresponded to the NOR sites in the genomes of all species. The 5S rDNA sequences were located near the centromeric region in a medium-sized pair in all Asian species, except *C. chitala*, in which 5S rDNA sites were observed on three different pairs, one co-localized with the 18S rDNA. In *X. nigri* 5S rDNA probes hybridized on two chromosome pairs, near to the centromeric region of a large chromosome and to the telomeric region of a medium-sized chromosome. In the karyotypes of *P. afer* and *C. chitala*, the 5S rDNA probes hybridized on three chromosome pairs, in one of them, the 5S probes were co-localized with the 18S rDNA sequences (Figure 4).

### 3.3. Chromosome Mapping of Microsatellite Motif Sequences

**Figure 6:**
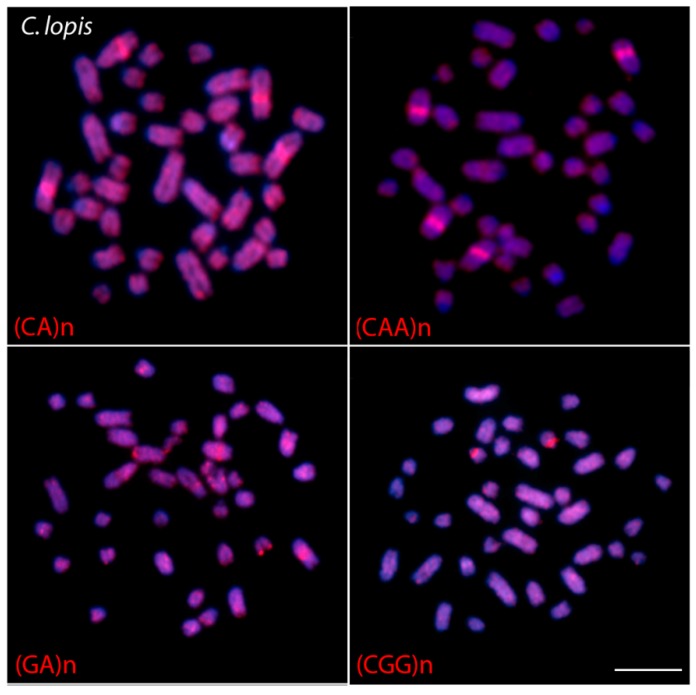
Metaphase chromosomes of *C. lopis* hybridized with different labeled microsatellite-containing oligonucleotides. Scale bar = 5 µm.

The genomes of all species showed the same hybridization pattern of microsatellites motifs: (CA)_15_ had a dense accumulation on chromosomes, in contrast with (GA)_15_ and (CAA)_10_ that had faint hybridization signals, and CGG10, that showed strong hybridization only near the 18S site (Figures S1 and S2). In particular, the genome of *C. lopis* showed a high accumulation of the microsatellites (CA)_15_, (GA)_15_ and (CAA)_10_ on the chromosomal region harboring the ITS (Figure 6).

### 3.4. Feature Annotation of Diversity Arrays Technology Sequencing Markers

To extract genomic features from DArTseq alleles, it was used BLAST searches against three collections of Teleostei sequences from RefSeq/NCBI databases, that is (1) “Osteoglossiformes RefSeq NUC” (which contains 48,195 nucleotide sequences references (41,432 mRNA and 6763 genomic) from 23 species (41,173 of them belong to *Scleropages formosus*)), (2) “Osteoglossiformes RefSeq PTN” (which contains 41,731 protein sequences references from 239 species (41,445 of sequences belong to *S. formosus*)), and (3) “Teleostei RefSeq mRNA” (a customized Teleostei collection which contains 373,153 mRNA sequences references from six Teleostei fishes). Due to the large size of full Teleostei nucleotide collection at NCBI (over than 13.7 million sequences) a refined collection for Teleostei was built selecting six representative species from six different orders with the highest number of sequences at RefSeq mRNA NCBI database.

**Figure 7:**
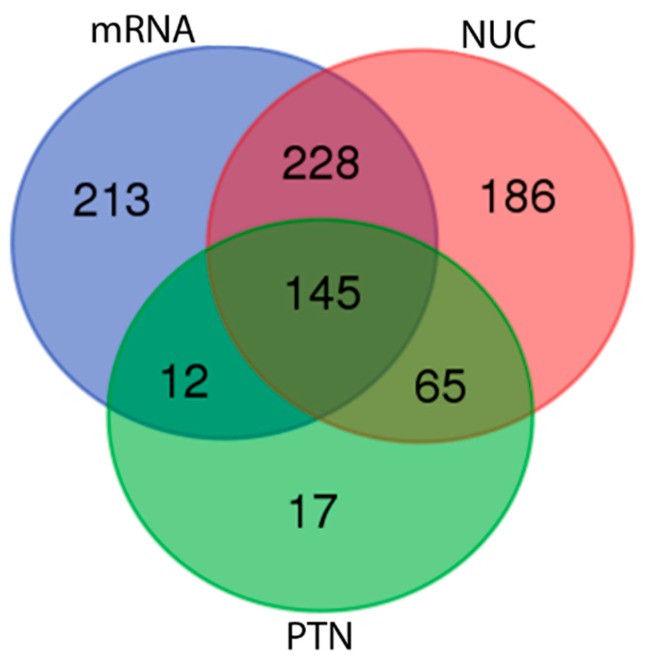
Venn diagram of BLAST hits for 1537 DArTseq allele sequences against three RefSeq fish collections. Details for sequence collections can be viewed in Table 2. NUC: Osteoglossiformes genomic sequences. PTN: Osteoglossiformes protein sequences. mRNA: Teleostei mRNA sequences.

**Figure 8:**
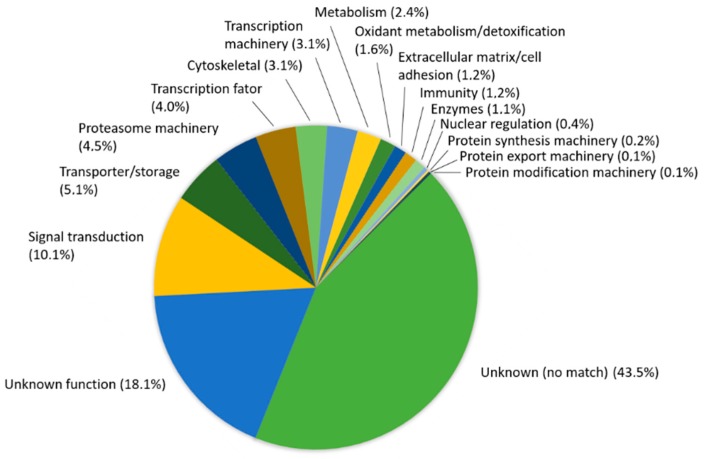
Functional annotation of 868 DArTseq allele sequences through BLAST hit description, calculated from Table S1. Allele sequences were compared to three collections of Teleostei sequences from RefSeq/NCBI database, Teleostei mRNA (373,153 mRNA sequences from six species), Osteoglossiformes Nucleotide (48,195 genomic/mRNA sequences from 23 species) and Osteoglossiformes Protein (41,731 protein sequences from 239 species). BLAST searches used a cut-off *E*-value (Expected value) lower than 0.1. Functional classification of allele sequences was manually curated based on description of best matches against the three RefSeq collections above.

About 40% of the allele sequences presented BLASTn hits against the nucleotide databases (Osteoglossiformes RefSeq NUC and Teleostei RefSeq mRNA), whereas a small portion of them (15.5%) matched against the protein collection (Osteoglossiformes RefSeq PTN) (Table 3), calculated from (Table S3), suggesting that the majority of the allele sequences now described are novelties, because no matches were found against over 450,000 available sequences. Most of allele sequences matched in at least two RefSeq collections and only 145 (9.4%) out of 1,1537 sequences presented BLAST results for the three sequence collections, as showed in the Venn diagram (Figure 7). No function could be assigned for approximately 18.1% of the 868 allele sequences without any match, being signal transduction, transporter/storage, proteasome machinery, and transcriptional factor the most prevalent functional classes (Figure 8).

### 3.5. Comparative Analyses Using Diversity Arrays Technology Sequencing Data

The DArTseq genotyping output (Table S2) consists of a matrix of “absence/presence of allele” (0/1) for each notopterid species for a given allele ID (rows), in which SNP calling relies on different statistical measures. An overview of the genotyping data showed that from 1537 SNP alleles found in six Notopteridae fishes, 57% of them showed transition type mutations, 88% presented only one SNP along the sequence, and 19% were found in heterozygosity (Table 4), calculated from (Table S2).

Overall data quality was high, as the consistency of average allele calling was above 99%, a very good result given the complexity of such task in case of such evolutionary distant material. This evolutionary distance resulted also in varying call rates among the samples tested: while the *C. lopis*, *C. ornata*, and *N. notopterus* were called for around 90% of loci, the other species (especially *C. chitala*) were called less efficiently, almost certainly due to the divergence at the restriction enzyme recognition sequences resulting in elimination of many fragments from genomic representations.

**Figure 9:**
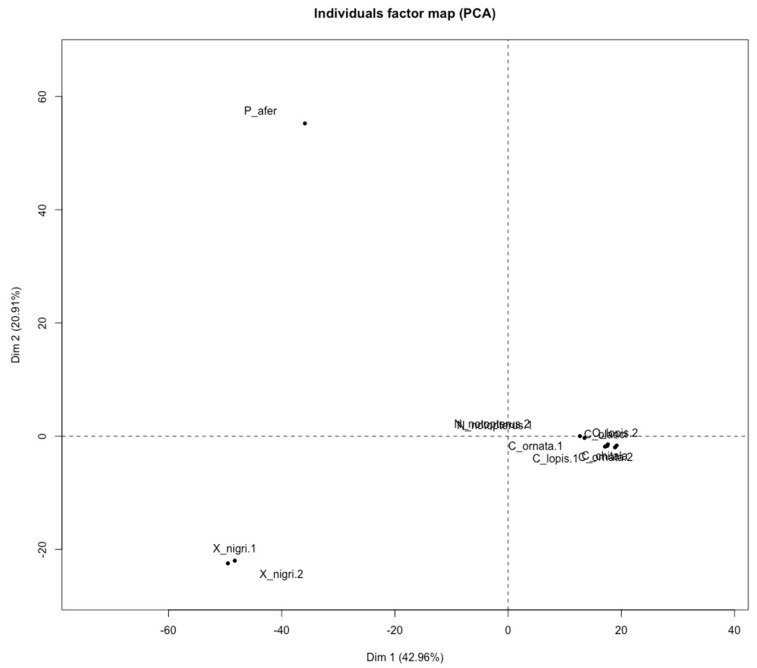
Principal component analyses of SNP data in DArTseq alleles found in the seven Notopteridae species analyzed (*Chitala ornata, C.blanci, C, lopis, C. chitala, N. notopterus, P. afer and X. nigri*). Individuals factor map using 3074 alleles (reference and alternative alleles). The notopterid specimens and presence/absence SNP were structured as observations (individual) and variable, respectively, as input matrix data.

**Figure 10:**
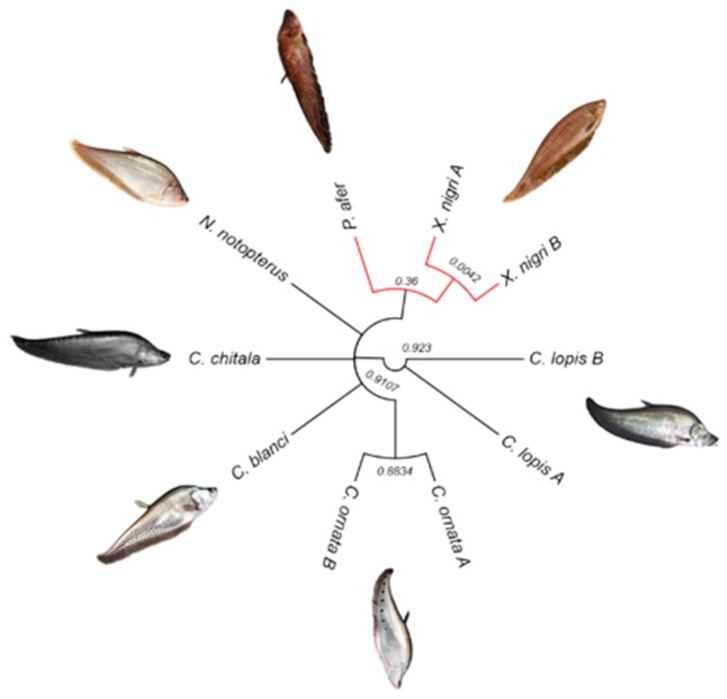
Dendogram generated by Bayesian combined molecular and chromosomal data from the notopterid family. Red branches represent species from Africa. Numbers in the nodes represents the Bayesian posterior probabilities.

Principal component analyses using the whole dataset of SNP files (3074 alleles, reference and alternative sequences) showed that Notopteridae species clustered according to their geographical distribution (Figure 9). In fact, the same results were observed when the relationships among notopterid species were reconstructed using the DArTseq data associated with chromosomal characters (Figure 10).

## 4. Discussion

### 4.1. Chromosomal Evolution in Notopteridae

The sum up of several characteristics, such as wide geographic distribution, ancient evolutionary origin, and adaptations to diverse environments, makes Osteoglossiformes a significant group for systematic and evolutionary studies [52,53,54]. However, cytogenetic and genomic data are still scarce for these fishes, making it difficult to infer on their evolutionary biology. Here, we used multipronged analyses, including chromosomal and genomic approaches, to evaluate the genetic relationships between African and Asian Notopteridae species, and to infer the impact of the continental drift on their biodiversity.

Karyotype descriptions for six of the species under study corroborated with our results, except *P. afer*, which had 2n = 50 (2m + 2sm + 46a), and not 2n = 34 (4sm + 30a) described by [22]. Actually, the three *P. afer* individuals examined by [22] were of unknown origin, which hampers conclusions about differences of data. Therefore, we cannot exclude the incidence of chromosomal variation among *P. afer* populations, or even that another *Papyrocranus* species, *P. congoensis*, has been analyzed instead. In fact, a similar inconsistency was also found for another osteoglossiform species, butterfly fish, *Pantodon buchholzi*, in which 2n = 46 occurs in individuals from the lower Niger River basin [19], compared to uncertain chromosome numbers (2n = 46 or 48) previously reported by [22] for individuals of unknown origin. Interestingly, the karyotype of *P. afer* possess 2n = 50, i.e., chromosome number hypothesized as basal and/close to 2n for teleosts [55], and as compared to karyotypes of all other notopterids, also contains bi-armed elements in karyotype. Three likely complementary scenarios might be responsible for such a derived karyotype pattern. The recent molecular phylogenetic and paleontological data indicate that clade of African notopterids is more basal to their Asian sister clade [4], and hence, a longer evolutionary timespan enabled karyotype differentiation. This, however, contradicts the karyotype pattern of *X. nigri*, which is identical with majority of other species. Another possibility lies in the different trajectory in karyotype differentiation in *Papyrocranus*. While likely common ancestor of notopterids experienced reduction of 2n via tandem fusions to 2n = 42 (and further in *C. lopis* to 2n = 38), in *Papyrocranus* lineage, 2n remained unchanged but associated with intrachromosomal rearrangement of two chromosomes pairs, resulting in bi-armed elements. Examination of another species, *P. congoensis*, will thus answer this question, leaving two possibilities where karyotype and 2n will be (i) the same as in *P. Afer*; or (ii) correspond to all other notopterids. Karyotype of *C. lopis* with 2n = 38 represents another exception to uniform 2n = 42 and identical karyotypes within Notopteridae (Figure 2). The likely scenario indicates tandem fusions responsible for this reduction of 2n. Actually, ITS were observed in the first and the third chromosomal pairs in karyotype of *C. lopis* (Figure 5), supporting such fusions events explaining 2n = 38 from 2n = 42 found in the other *Chitala* species.

A considerable fraction of the eukaryotic genome consists of repetitive DNA sequences, which include multigene families, satellites, microsatellites, and transposable elements (TEs) [56]. Although the rDNA multigene families have been reported in a small number of teleosts, their distribution in only one chromosome pair represents the most frequent condition [57]. In Osteoglossiformes, only genomes of *Arapaima gigas*, *P. buchholzi*, and some Mormyridae species had these genes already mapped. In all cases, the major 18S rRNA genes were confined to only one chromosome pair, but evidently not homologous among these species [58,59,60]. Our present data also revealed the same pattern, i.e., 18S rDNA sites located in a single chromosome pair, fully corresponding with Ag-NOR sites as well as with GC-rich heterochromatin. This correspondence between cytogenetically detectable GC-rich DNA and sites of major rDNA sites have been documented in all major clades of actinopterygian fishes [61,62,63,64,65], except Acipenseriformes [66], and this character appears likely evolutionary conserved. Our present results for notopterids confirm this pattern. In contrast to 18 rDNA sites, the 5S rRNA genes had a more variable distribution pattern, showing differential distributions of number and location among species, as well as their co-localization with 18S rDNAs in *C. chitala* and *P. afer* (Figure 4). In fact, non-syntenic organization for both rDNA classes is considered the common pattern for Teleosts, with few exceptions [67,68,69,70,71,72,73]. However, our present finding of a syntenic organization of both rDNA clusters in such an ancient teleost lineage does not fit this previous point of view.

Microsatellites, or simple sequence repeats, are substantial and hypervariable components of the genome, made up of tandemly short DNA motifs [74]. Such repeats are usually associated with the heterochromatic regions of fish genomes and particularly accumulated in the sex chromosomes (reviewed in [75]). Among Notopteridae, the (CA)_15_ and (GA)_15_ dinucleotide sequences accumulated in the subtelomeric regions of all chromosomes, while the other microsatellites showed a dispersed distribution over chromosomes, including euchromatic and heterochromatic regions, despite some accumulation of (GAA)_10_ in the telomeric regions. Contrasting with the present data, only the (GAA)_10_ microsatellite produced a clear and abundant hybridization pattern in the pericentromeric/telomeric heterochromatin of some Mormyridae species [60].

Except for the differential distribution of some repetitive DNA classes, the karyotypes and some other chromosomal markers of the Notopteridae species did not show significant differentiation. On the contrary, five out of seven examined species possessed the same 2n and karyotype structures, interestingly, both in representatives of African and Asian clades. The age of split of both clades was estimated in broad time span −133 Mya [10] and 90–188 Mya (such large intervals depend on the dating of crown Teleostei) [4], and geological separation of Africa and Madagascar + India to 135 Mya [76,77]. In result, African and Asian notopterids are separated each from other around 100 Mya, and they still retain the same and/or nearly the same karyotype pattern, evidently, as their common ancestor. Cytotaxonomy of recent notopterids thus provides another strong example of chromosomal stasis among teleostean fishes (e.g., [78]). The evolutionary forces beyond such remarkable stasis in a number of (not only) teleostean groups are largely out of the present paper.

### 4.2. Genetic Variability among Notopterids

The development and improvement of large-scale genotyping-by-sequencing techniques allowed high-resolution analysis using SNP for phylogenetic, genetic diversity, and genomic selection in non-model organism investigations [79,80,81,82]. Our results demonstrate that DArT markers were very informative, as they provided high PIC values, call rate, and scoring reproducibility. They showed a robust efficiency in the analysis of genetic diversity among all Notopteridae species here analyzed. Results were highly consistent with the chromosomal, geographical, and historical data, lighting the evolutionary diversification of Notopteridae. The PCA showed a pattern of genetic differentiation among the samples, which allowed their clusterization into two major groups according to their geographical distribution: (i) the African species (*X. nigri* and *P. afer*); (ii) and the Asian species (*N. notopterus* and all *Chitala* species) (Figure 9).

Functional annotation of DArTseq alleles were categorized after BLAST searches against three collections of Teleostei sequences from RefSeq/NCBI databases, and grouped into 15 broad categories of biological functions (Figure 10). They were assigned according to the involvement of a gene or a protein in a cellular process or pathway, as opposed to its participation in a specific binding or catalytic functions. Since there are no available sequenced genomes for any Notopteridae species, a large proportion of DArTseq alleles matched to “unknown proteins” or proteins with “unknown function”. The remaining alleles were associated with some broad categories, including signal transduction, transporter/storage, proteasome machinery, transcriptional factor, and cellular communication/signal transduction (Figure 10).

Notably, all *Chitala* species presented a relatively higher number of rare alleles per accession as compared to other groups (Table S4). Maybe the unique allelic diversity of *Chitala* species is associated with the adaptation of this species to the South Asian environment after their arrival in the Asian continent at ~55 Mya [10]. In this sense, comparative analyses with the diversity profile of other Osteoglossiformes species inhabiting distinct geographical regions will probably generate useful information regarding the evolutionary history and adaptation of this ancient fish group.

### 4.3. Hypotheses on Biogeographical History of Notopteridae in Light of Our Data

**Figure 11:**
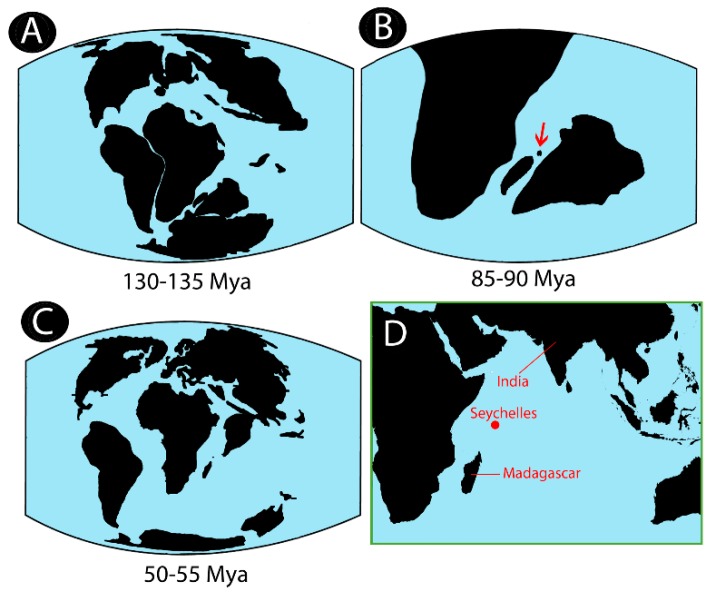
Illustration of key steps for Notopteridae biogeography: (**A**) Split of India from the Gondwana continent; (**B**) Geographic distribution of Seychelles–India–Madagascar–Africa; (**C**) Arrival of India subcontinent to Eastern Asia; (**D**) Actual location of Seychelles, India, and Madagascar.

Our results demonstrate two major notopterid clades based on genomic and chromosomal features: the African and the South Asia species groups, where *C. chitala* presenting intermediate characteristics between them (Figure 4). Remarkably, *C. chitala* has the narrowest distribution inside the Asian continent, restricted to India only [7], indicating that India probably represented the migration route for notopterids to Asia. Nowadays, the most accepted hypothesis to explain their dispersal to the Asian continent is the “Out-of-India” hypothesis, where lineages would have directly diverged by the impact of the African continent and India subcontinent separation at approximately 135 Mya [76,83]. However, recent molecular phylogenetic studies do not completely support this hypothesis [4]; using nuclear and mitogenomic markers and calibrating the analyses with fossil records, it is estimated that the time of divergence between these lineages occurred between a range of 80–120 Mya. For Notopteridae, the weakness of this hypothesis, i.e., maintenance of gene flow for at least 15 million years after the separation of the continental masses, is that notopterids are primarily freshwater fishes, with absence of marine fossils. With the exception of *N. notopterus*, that can occasionally sail in brackish waters, none of the other extant notopterids are able to tolerate salt water [7]. Indeed, recent studies pointed out alternatives, probably allowing gene flow between populations after their separation from Gondwana, without the need for transmarine migration. Between 118 and 130 Mya ago, Madagascar had already reached its current geographic position relative to Africa, but fossil records support the occurrence of connections between India and Madagascar as late as 80 Mya before [84,85,86,87,88]. The missing link for this scenario could be Seychelles and its particular geological history. Seychelles is an archipelago formed by small 155 islands located in the Indian Ocean northeast off Madagascar. That harbored an intense volcanic activity that could have created temporary landmasses bridges allowing interconnections among Africa–Madagascar–India subcontinent until ~80 Mya [83,86]. Additionally, several plateaus, banks, and fracture zones may have emerged periodically from the ocean facilitating biotic interchanges between these regions [89]. Such land bridges may have served as “stepping stones” for biota exchanges between India and Madagascar, a hypothesis known as “Lemurian Stepping-stones” [90,91,92], thus explaining the distribution of several taxa, such as lemuriform primates, snakes, frogs, iguanid lizards, and plant groups [84]. Further, the complete separation of India subcontinent and Madagascar occurred around 85–90 Mya [93] (Figure 11).

In fact, East Africa is an important source of Seychelles’ colonizers, also for freshwater organisms, such as evidenced for freshwater crabs [94]. Therefore, when the Madagascar–Seychelles–India continent rifted from Gondwana, it probably held a diversity of life forms, including several Notopteridae lineages. In addition, during the Mesozoic and Cenozoic, Indian North and East Africa were flooded by shallow seas that suffered several retractions in the Eocene, enabling the migration possibility between these continental masses, even after their separation of the Gondwana [83]. These would have allowed gene flow until 85–90 Mya, which matches with the proposed divergence time between African and Asian notopterids, that occurred around 80–120 Mya [4].

As well as Notopteridae, several other primary freshwater fishes show an African–Asian distribution (e.g., Bagridae, Schilbeidae, Clariidae, Aplocheilidae, Mastacembelidae, Cyprinids, Anabantidae, and Channidae) [8]. Remarkably, most of them have conspicuous karyotype diversity, resulting from millions of years of restricted gene flow between African and Asian clades [95]. Otherwise, despite the significant genetic diversity and the longtime of divergence, the majority of the notoperids retain the same karyotype features at the macrostructural level. Why are the evolutionary relationships of the Notopteridae so divergent at the chromosomal and genomic levels? What has shaped its particular mode of evolution? Up to now, there are no convincing answers clarifying such intriguing questions.

## Figures and Tables

**Table 1:** Review of available data on 2n, karyotypes and fundamental number (FN) in the Notopteridae family. (m) metacentric; (sm) submetacentric; (st) subtelocentric; and (a) acrocentric chromosomes.

Species	2n	Karyotype	FN	Reference	Locality
*Chitala chitala*	42	42a	42	Present study	Song Khram basin, Thailand
*C. chitala*	48	12m + 36a	60	[21]	India, Delhi, River Jumna
*C. chitala*	42	42a	42	[22]	Not known
*C. chitala*	42	42a	42	[23]	Aquarium trade
*Notopterus notopterus*	42	42a	42	Present study	Chi and Mekong basins, Thailand
*N. notopterus*	42	42a	42	[24]	India, Kurukshetra
*N. notopterus*	42	42a	42	[25]	Thailand
*N. notopterus*	48	12m + 36a	60	[21]	India, Delhi, River Jumna
*N. notopterus*	42	42a	42	[26]	Not known
*N. notopterus*	42	42a	42	[27]	Thailand
*N. notopterus*	42	42a	42	[28]	Thailand, Chi Basin.
*Chitala ornata*	42	42a	42	Present Study	Chi and Mekong basins, Thailand
*C. ornata*	42	42a	42	[27]	Thailand, Chi Basin
*C. ornata*	42	42a	42	[29]	Thailand, Chi Basin
*C. blanci*	42	42a	42	Present Study	Song Khram basin, Thailand
*C. blanci*	42	42a	42	[27]	Thailand, Chi Basin
*C. lopis*	38	38a	38	Present study	Song Khram basin, Thailand
*Papyrocranus afer*	34	4sm + 30a	38	[22]	Africa
*P. afer*	50	2m + 2sm + 36a	54	Present study	Oluwa River, Nigeria
*Xenomystus nigri*	42	42a	42	[22]	Africa
*X. nigri*	42	42a	42	Present Study	Oluwa River, Nigeria

**Table 2:** Collection sites of the Notopteridae species and number of individuals analyzed in this study.

Species	Sampling Sites	N
*Chitala blanci*	Song Khram basin, Thailand	(04 ♀; 04 ♂)
*Chitala chitala*	Ganges river, India	(05 ♀; 04 ♂)
*Chitala lopis*	Song Khram basin, Thailand	(12 ♀; 06 ♂)
*Chitala ornate*	Chi and Mekong basins, Thailand	(09 ♀; 07 ♂)
*Notopterus notopterus*	Chi and Mekong basins, Thailand	(06 ♀; 04 ♂)
*Papyrocranus afer*	Oluwa River, Nigeria	(19 ♀; 21 ♂)
*Xenomystus nigri*	Oluwa River, Nigeria	(13 ♀; 24 ♂)

**Table 3:** Basic local alignment search tool (BLAST) searches of DArTseq (diversity arrays technology sequencing) allele sequences against collections of fish sequences from RefSeq/NCBI database.

Sequence Collections Retrieved from NCBI	Number of Representative Species	Number of Sequences ^c^	Number of DArTseq Alleles with BLAST Hits ^a^
Teleostei RefSeq mRNA	6 ^b^	373,153	619 (40%)
Osteoglossiformes RefSeq Nucleotide	23 ^d^	48,195	625 (41%)
Osteoglossiformes RefSeq Protein	239 ^e^	41,731	238 (16%)

^a^ Percentage in parenthesis referring to 1537 allele sequences searched against each collection. ^b^ *Salmo salar*, *Sinocyclocheilus rhinocerous*, *Oreochromis niloticus*, *Esox lucius*, *Poecilia formosa* and *Ictalurus punctatus*. ^c^ Collection comprised by genomic and mRNA sequences. ^d^ Most sequences (48,173) from *Scleropages formosus*. ^e^ Most sequences (41,445) from *Scleropages formosus*.

**Table 4:** General characteristics of single-nucleotide polymorphism (SNP) data for 1537 alternative alleles found in six Notopteridae species.

Type of Mutation	Count	Percentage
Transition	882	57%
Transversion	655	42%
Unique SNP in an allele sequence	1201	88%
Multiple SNP in an allele sequence		
2 SNP	143	10.50%
3 SNP	10	0.70%
4 SNP	1	0.07%
5 SNP	1	0.07%
11 SNP	1	0.07%
Alleles found in Heterozygosity	287	19%

## Supplementary Materials

The following are available online at http://www.mdpi.com/2073-4425/9/6/306/s1, Figure S1: Metaphase chromosomes of *Chitala* species hybridized with different labeled microsatellite-containing oligonucleotides. Scale bar = 5 µm, Figure S2: Metaphase chromosomes of *Notopterus notopterus*, *Xenomystus nigri*, and *Papyrocranus* after hybridization with different labeled microsatellite-containing oligonucleotides. Bar = 5 µm. Table S1: List of all SNP data generated by DArTseq. FreqHomRef represents frequency of homozygotes for reference allele (more common major allele), FreqHomSnp represents frequency of homozygotes for SNP allele (less common minor allele), and FreqHets represents frequency of heterozygotes, Table S2: Coded data matrix information using chromosomal characters from the present study. Karyotype number code, 2n = 42 = 0, 2n = 38 = 1, 2n = 50 = 2; Chromosomal arms code, Acrocentric = 0, Metacentric = 1; Telomeric Marker code, Terminal = 0, Interstitial = 1; 5S Marker; simple = 0, multiple = 1; 18S marker, short arm = 0, long arm = 1; 5S and 18S markers, no-sintenic = 0, sintenic = 1, Table S3: BLAST results of DArTseq alleles against customized collections of fish sequences from RefSeq/NCBI database. Column A indicates the allele ID according to Table S1. Column D indicates functional annotation abbreviation based on best matches. Columns G, Z, and AL display the names of best matches results for Teleostei mRNA, Osteoglossiformes nucleotide, and Osteoglossiformes protein sequence collections, respectively. Columns H to Y, AA to AK, and AM to AY display BLAST statistics and sequence features for each best hit from a respective sequence collection, Table S4. Functional annotation of 868 DArTSeq allele sequences through BLAST hit description only for *Chitala* species.
