# Supplementary figures and images for: From Chromosomes to Genome: Insights into the Evolutionary Relationships and Biogeography of Old World Knifefishes (Notopteridae; Osteoglossiformes)
Source: Genes (Basel). 2018 Jun 19;9(6):306. doi: 10.3390/genes9060306 (PMC6027293; doi:10.3390/genes9060306)

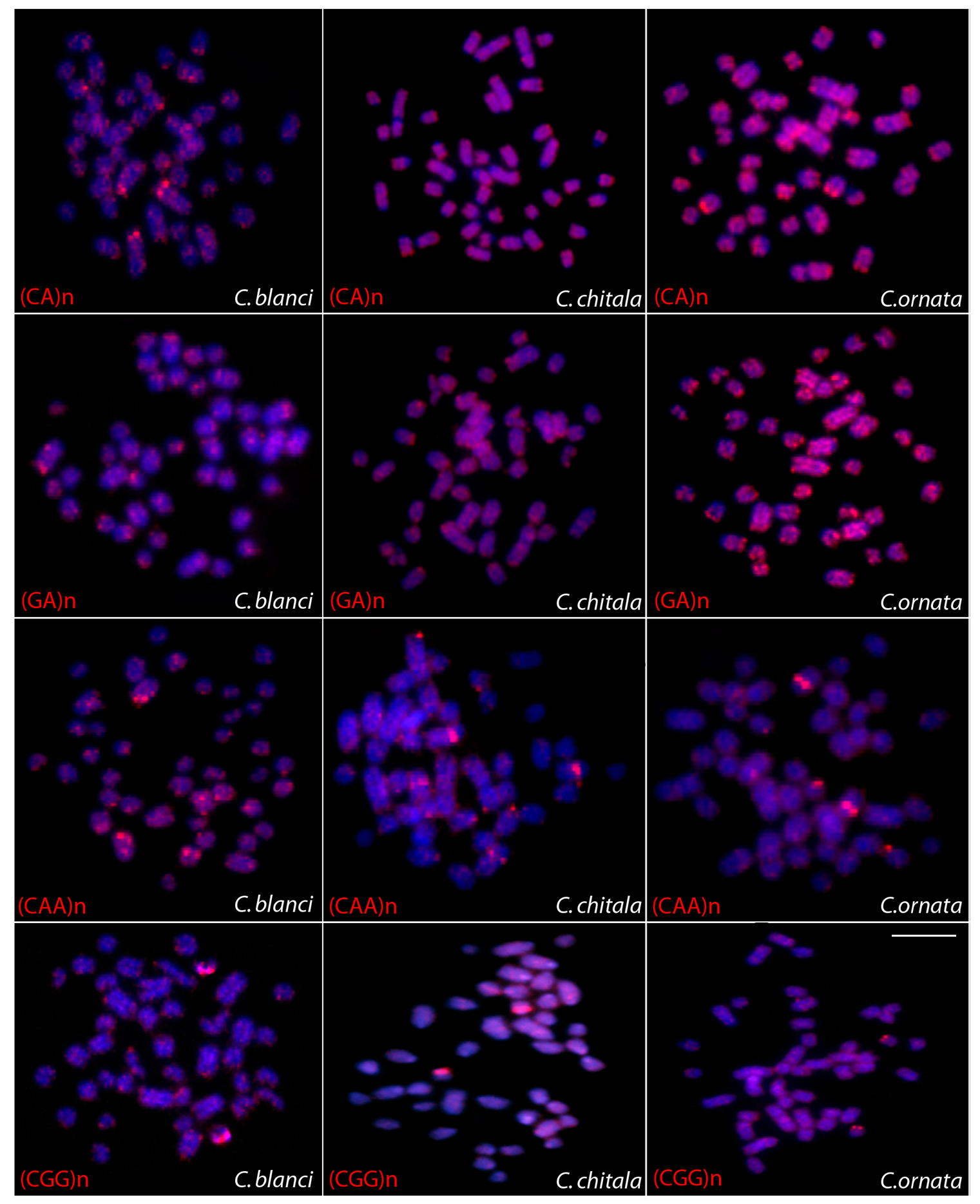

Supplement: Supplementary file 1 [file genes-09-00306-s001.zip › Supplementary Figure 1.tif]

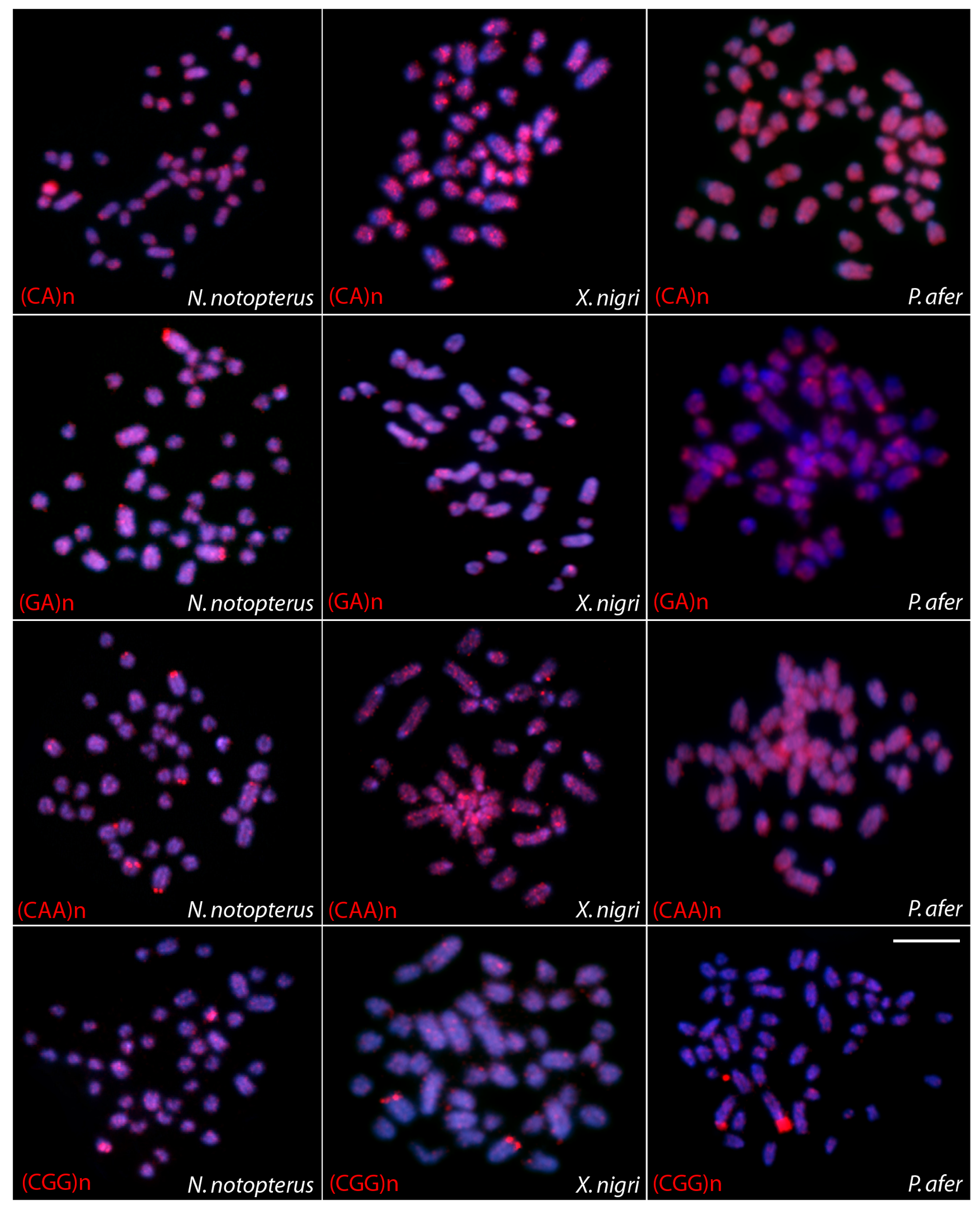

Supplement: Supplementary file 1 [file genes-09-00306-s001.zip › Supplementary Figure 2.tif]
