# Supplementary material for: From Chromosomes to Genome: Insights into the Evolutionary Relationships and Biogeography of Old World Knifefishes (Notopteridae; Osteoglossiformes)
Source: Genes (Basel). 2018 Jun 19;9(6):306. doi: 10.3390/genes9060306 (PMC6027293; doi:10.3390/genes9060306)
Supplement: Supplementary file 1 [file genes-09-00306-s001.zip › Supplementary Table 2.doc]

**S Table 2.** Coded data matrix information using chromosomal characters from the present study. Karyotype number code, 2n = 42 = 0, 2n = 38 = 1, 2n = 50 = 2; Chromosomal arms code, Acrocentric = 0, Metacentric = 1; Telomeric Marker code, Terminal = 0, Intersticial = 1; 5S Marker; simple = 0, multiple =1; 18S marker, short arm = 0, long arm = 1; 5S and 18S markers, no-sintenic = 0, sintenic =1.

| **Specie** | **Karyotype number** | **Chromosomal arms** | **Telomeric marker** | **5S marker** | **18S marker** | **5S and 18S markers** |
| --- | --- | --- | --- | --- | --- | --- |
| *C. lopis* | 1 | 0 | 1 | 0 | 0 | 0 |
| *C. ornata* | 0 | 0 | 0 | 0 | 0 | 0 |
| *N. notopterus* | 0 | 0 | 0 | 0 | 0 | 0 |
| *P. afer* | 2 | 1 | 0 | 1 | 0 | 1 |
| *C. chitala* | 0 | 0 | 0 | 1 | 0 | 1 |
| *C. blanci* | 0 | 0 | 0 | 0 | 0 | 0 |
| *X. nigri* | 0 | 0 | 0 | 1 | 1 | 0 |
