# Supplementary material for: From Chromosomes to Genome: Insights into the Evolutionary Relationships and Biogeography of Old World Knifefishes (Notopteridae; Osteoglossiformes)
Source: Genes (Basel). 2018 Jun 19;9(6):306. doi: 10.3390/genes9060306 (PMC6027293; doi:10.3390/genes9060306)
Supplement: Supplementary file 1 [file genes-09-00306-s001.zip › Supplementary Table 4.doc]

**S Table 4.** Functional annotation of 868 DArT-Seq allele sequences through BLAST hit description only for *Chitala* species.

| **Functional class** | **Abbreviation^a^** | **Number of allele sequences** | **%** |
| --- | --- | --- | --- |
| Unknown function | uk | 155 | 57,8 |
| Signal transduction | st | 19 | 7,08 |
| Unknown (no match) | uknm | 14 | 4,96 |
| Proteasome machinery | prot | 16 | 5,97 |
| Cytoskeletal | cs | 13 | 4,85 |
| Transporter/storage | tr/storage | 12 | 4,47 |
| Oxidant metabolism/detoxification | detox/ox | 9 | 3,35 |
| Transcription factor | tf | 7 | 2,61 |
| Enzymes | enz/protease | 7 | 2,61 |
| Immunity | imm | 4 | 1,49 |
| Transcription machinery | tm | 3 | 1,11 |
| Metabolism | met | 3 | 1,11 |
| Extracellular matrix/cell adhesion | ext/mat | 3 | 1,11 |
| Nuclear regulation | nr | 3 | 1,11 |
| Metabolism, carbohydrate | met/carb | 1 | 0,37 |
| **TOTAL** |  | 268 |  |

a. Abbreviation as in column “class” of Supplementary Table X2.
